# Effect of Low-Level Laser Therapy on Implant Osseointegration and Peri-Implant Clinical Outcomes: A Prospective Observational Study

**DOI:** 10.7759/cureus.114626

**Published:** 2026-08-16

**Authors:** Pritha Negi, Aishla Jain, Sushma J, Lakshmi Sailaja Akella, Sanpreet S Sachdev, Abida Parveen

**Affiliations:** 1 Department of Prosthodontics, Crown and Bridge, K.M. Shah Dental College and Hospital, Sumandeep Vidyapeeth (Deemed to be University), Vadodara, IND; 2 Department of Dentistry, All India Institute of Medical Sciences, Jodhpur, IND; 3 Department of Prosthodontics, Rajarajeswari Dental College and Hospital, Bangalore, IND; 4 Department of Prosthodontics, Gandhi Institute of Technology and Management (GITAM) Dental College and Hospital, Visakhapatnam, IND; 5 Department of Oral Pathology and Microbiology, Bharati Vidyapeeth (Deemed to be University) Dental College and Hospital, Navi Mumbai, IND; 6 Department of Orthodontics, Maharishi Markandeshwar College of Dental Sciences and Research, Ambala, IND

**Keywords:** dental implants, implant stability, low-level laser therapy, marginal bone loss, osseointegration

## Abstract

Introduction: Low-level laser therapy (LLLT) has been proposed as an adjunctive modality to enhance osseointegration and improve peri-implant healing after dental implant placement. However, evidence regarding its clinical effectiveness remains inconclusive. This study evaluated the effects of adjunctive LLLT on implant stability, marginal bone remodeling, peri-implant probing depth, and postoperative complications over a 12-month follow-up period.

Materials and methods: A prospective observational clinical study was conducted on 80 dental implants placed in patients requiring single-tooth implant rehabilitation. Following standardized implant placement, all participants received LLLT using an Epic X diode laser (BIOLASE, Inc., Foothill Ranch, CA, USA) immediately after surgery and on postoperative days 3, 7, and 14. Implant stability was assessed using resonance frequency analysis, whereas radiographic marginal bone levels and peri-implant probing depth were evaluated at prosthetic loading, at six months, and at 12 months. Data were analyzed using repeated-measures analysis of variance with Bonferroni-adjusted post hoc testing.

Results: Implant stability improved significantly during follow-up, with the mean implant stability quotient increasing from 67.98 ± 4.02 at implant placement to 74.28 ± 4.54 at 12 months (p < 0.001). Marginal bone loss increased gradually from 0.19 ± 0.08 mm at prosthetic loading to 0.55 ± 0.16 mm at 12 months but remained within clinically acceptable limits (p < 0.001). Mean peri-implant probing depth increased modestly from 2.13 ± 0.47 mm to 2.52 ± 0.61 mm during follow-up (p < 0.001). No complications were observed in 71 (88.8%), 69 (86.2%), and 64 (80.0%) implants at prosthetic loading, six months, and 12 months, respectively.

Conclusion: Adjunctive LLLT was accompanied by progressive improvement in implant stability, favorable peri-implant tissue healing, acceptable marginal bone remodeling, and a low incidence of postoperative complications during the 12-month follow-up period. However, in the absence of a control group, these outcomes cannot be attributed specifically to LLLT. Larger randomized controlled trials with longer follow-up periods are required to determine whether LLLT provides additional clinical benefits beyond conventional implant healing.

## Introduction

Successful osseointegration remains the cornerstone of long-term dental implant therapy [1]. Although contemporary implant systems demonstrate high survival rates, the biological events occurring during early healing largely determine implant stability, peri-implant bone preservation, and timing of prosthetic rehabilitation [2]. Primary implant stability is achieved through mechanical engagement with the surrounding bone, whereas secondary stability develops through bone remodeling and new bone formation at the implant-bone interface [3]. Numerous local and systemic factors influence this healing process, prompting continued interest in adjunctive therapies capable of accelerating osseointegration while minimizing postoperative complications [1,4,5].

Low-level laser therapy (LLLT), also referred to as photobiomodulation therapy, has emerged as a non-invasive adjunctive treatment that modulates cellular activity without causing thermal tissue damage. By stimulating mitochondrial cytochrome c oxidase, LLLT enhances adenosine triphosphate production, promotes osteoblastic proliferation and differentiation, increases collagen synthesis, improves angiogenesis, and reduces inflammation [6]. These biological effects may contribute to faster bone healing, improved implant stability, and enhanced soft-tissue repair [7]. Furthermore, the analgesic and anti-inflammatory properties of LLLT may improve patient comfort during the postoperative healing period [8].

Despite increasing clinical interest, available evidence regarding the effectiveness of LLLT in implant dentistry remains inconsistent. Differences in laser wavelength, power output, energy density, irradiation protocol, implant systems, and outcome assessment methods have resulted in heterogeneous findings [7,9]. Consequently, further well-designed prospective clinical studies using standardized treatment protocols and longitudinal follow-up are required to clarify the clinical benefits of LLLT in implant healing and osseointegration.

This study aimed to prospectively evaluate the clinical course of osseointegration and peri-implant tissue health in dental implants receiving adjunctive LLLT. The primary objective was to assess longitudinal changes in implant stability using resonance frequency analysis. The secondary objectives were to evaluate radiographic marginal bone-level changes, measure peri-implant probing depth, determine healing time before prosthetic loading, and record postoperative complications over a 12-month follow-up period after prosthetic loading.

## Materials and methods

Study design, setting, duration, and ethical approval

This prospective observational clinical study was conducted in the Department of Prosthodontics, K.M. Shah Dental College and Hospital, Sumandeep Vidyapeeth (Deemed to be University), Vadodara, Gujarat, India, between December 2022 and November 2024, which included a 12-month follow-up period after prosthetic loading. The study protocol was approved by the Institutional Ethics Committee (SVIEC/Dent/2022/Nov/113). Written informed consent was obtained from all participants prior to enrollment.

Sample size estimation

The sample size was calculated using G*Power software version 3.1.9.7 (Heinrich Heine University Düsseldorf, Düsseldorf, Germany). Based on previously reported implant stability quotient (ISQ) values, an effect size of 0.32 was considered with a significance level (α) of 0.05 and a study power of 80% [10]. The minimum required sample size was estimated to be 79 implants, which was rounded to 80 implants.

Study participants

Eligible participants were screened according to predefined inclusion and exclusion criteria to ensure suitability for single-tooth implant-supported rehabilitation. Only individuals fulfilling all inclusion criteria (Table 1) were enrolled in the study.

**Table 1:** Inclusion and exclusion criteria for study participants.

Inclusion criteria	Exclusion criteria
Patients aged 18-65 years	Uncontrolled systemic diseases, pregnancy, and lactation
Requiring single-tooth implant-supported rehabilitation in either the maxillary or mandibular arch	Active periodontal disease
Adequate bone volume for implant placement without the need for simultaneous bone augmentation	Previous radiotherapy to the head and neck region
Good oral hygiene	History of bisphosphonate therapy
Primary implant stability of ≥30 Ncm at the time of implant placement	Heavy smoking (>10 cigarettes/day)

Implant placement and LLLT

All implant surgeries were performed by a single experienced implant surgeon under local anesthesia using a standardized surgical protocol. A total of 80 Touareg™-OS dental implants (Adin Dental Implant Systems Ltd., Afula, Israel) were used. The implants were tapered, spiral, internal-hex implants featuring the proprietary OsseoFix™ surface, a calcium-phosphate grit-blasted micro-roughened titanium surface designed to promote osseointegration. Implant diameters ranged from 3.5 to 5.0 mm, and lengths ranged from 8 to 13 mm, with implant dimensions selected according to the available bone volume, anatomical site, and prosthetic requirements. Of the 80 implants, 36 were placed in the maxilla and 44 in the mandible. Following crestal incision and full-thickness flap elevation, osteotomy preparation was completed according to the manufacturer's recommended drilling sequence under copious sterile saline irrigation. All implants were inserted with a minimum insertion torque of 30 Ncm, followed by placement of healing abutments or cover screws and wound closure using 4-0 non-resorbable sutures.

Immediately after implant placement, LLLT was performed using an Epic X diode laser (BIOLASE, Inc., Foothill Ranch, CA, USA) operating at a wavelength of 940 nm. Laser irradiation was delivered in continuous-wave mode with an output power of 0.5 W (500 mW) and an energy density of 4 J/cm². The laser was applied in the non-contact mode with the fiber tip maintained at approximately 2 mm from the mucosal surface. Irradiation was performed for 8 s at the buccal, lingual/palatal, mesial, and distal peri-implant surfaces, delivering approximately 4 J of energy per point. Laser therapy was administered immediately after surgery and was repeated on postoperative days 3, 7, and 14. All the participants received identical postoperative medications and oral hygiene instructions.

Clinical and radiographic assessment

**Figure 1:**
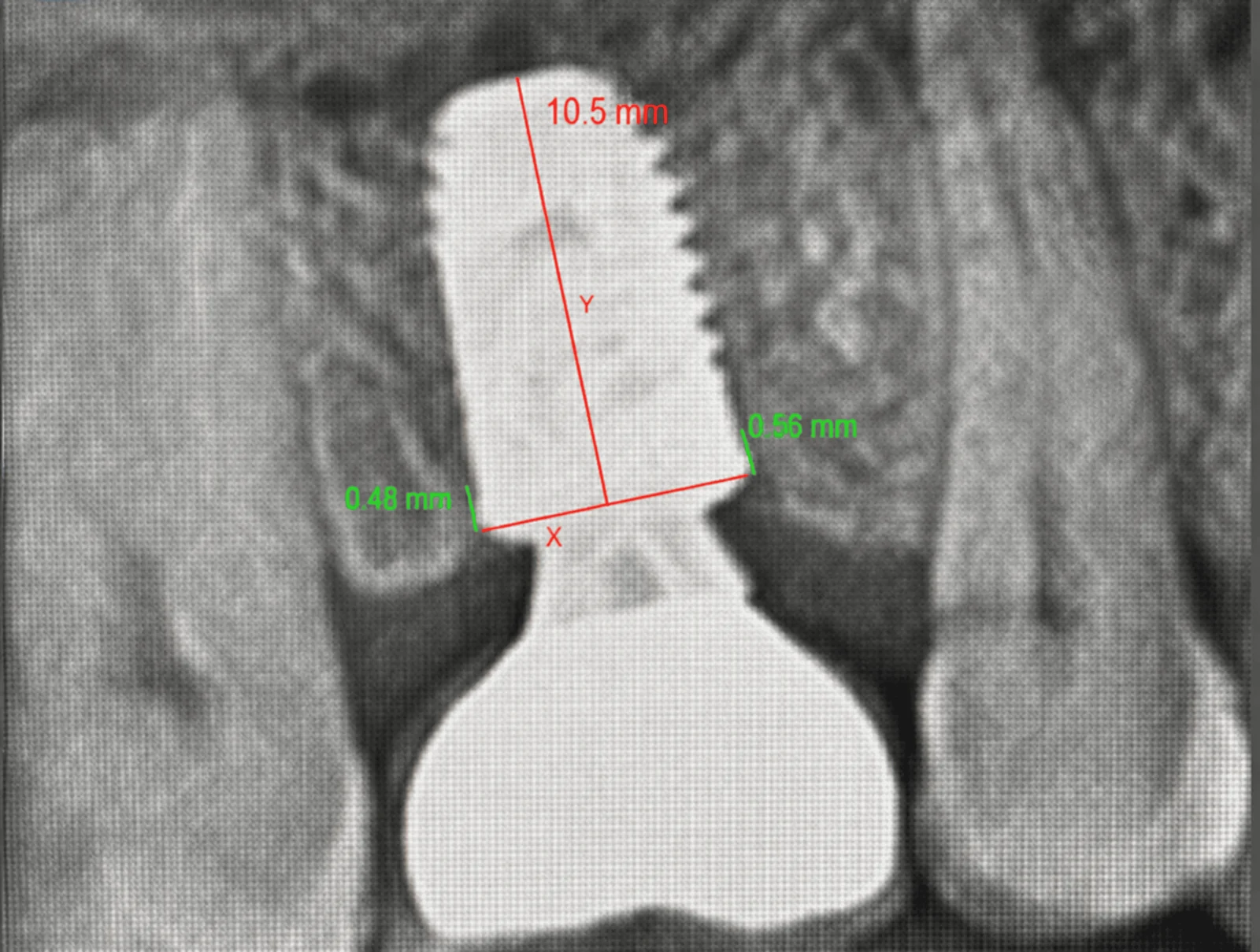
Measurement of radiographic bone loss on mesial and distal aspects of maxillary posterior implant. Green line: distance from implant platform to nearest apical location of bone to implant surface contact; red line (X): implant platform; red line (Y): implant length.

Implant stability was evaluated using resonance frequency analysis with an Osstell ISQ device (Osstell AB; Gothenburg, Sweden). Implant stability was assessed longitudinally at four clinically relevant time points: immediately after implant placement (baseline), at prosthetic loading, six months after prosthetic loading, and 12 months after prosthetic loading. These intervals were selected to characterize primary implant stability, stability following initial osseointegration, and subsequent changes during medium- and longer-term functional loading [11]. Radiographic marginal bone levels were assessed using standardized digital intraoral periapical radiographs obtained using the paralleling technique with customized positioning stents. The distance from the implant platform to the first bone-to-implant contact on the mesial and distal aspects was measured using ImageJ software version 1.54 (National Institutes of Health, Bethesda, MD, USA) (Figure 1). The peri-implant probing depth was recorded at four sites around each implant using a calibrated UNC-15 periodontal probe (Hu-Friedy Mfg. Co., LLC, Chicago, IL, USA), and the mean value was used for analysis. The healing time was defined as the interval between implant placement and prosthetic loading. Postoperative complications were recorded during the follow-up period.

Calibration and examiner reliability

Radiographic measurements were independently performed by two calibrated examiners (AJ and SJ) who were blinded to previous measurements. Before the study commenced, 20 radiographs not included in the final sample were evaluated twice at a one-week interval under standardized viewing conditions. Intra- and inter-examiner reliabilities were assessed using the intraclass correlation coefficient (ICC). Any disagreement greater than 0.2 mm was resolved through consensus before final data entry.

Statistical analysis

All participant information was anonymized using coded identification numbers before the statistical analysis. The coded dataset was independently analyzed by a statistician (AP). Statistical analysis was performed (IBM SPSS Statistics for Windows, Version 25.0, IBM Corp., Armonk, NY, USA). The normality of continuous variables was assessed using the Shapiro-Wilk test. Continuous data are expressed as mean ± standard deviation and 95% confidence intervals (CIs). Longitudinal changes in implant stability, marginal bone level, and peri-implant probing depth were evaluated using repeated-measures analysis of variance (ANOVA), followed by Bonferroni-adjusted post hoc comparisons. Categorical variables are expressed as frequencies and percentages. A two-tailed p < 0.05 was considered statistically significant.

## Results

The radiographic measurements demonstrated excellent reproducibility. The intra-examiner ICC was 0.94 (95% CI: 0.86-0.98), while the inter-examiner ICC was 0.91 (95% CI: 0.80-0.96), indicating excellent intra- and inter-examiner reliability. Eighty implants placed in eligible participants completed the 12-month follow-up and were included in the final analysis. The baseline demographic and clinical characteristics of the patients are summarized in Table 2. The mean age of the participants was 32.45 ± 12.34 years. The study population comprised 31 (38.7%) men and 49 (61.3%) women, and 12 (15.0%) participants reported a history of smoking. At implant placement, the mean insertion torque was 67.98 ± 4.02 Ncm, whereas the mean peri-implant probing depth and healing time at prosthetic loading were 2.13 ± 0.47 mm and 13.04 ± 1.78 weeks, respectively.

**Table 2:** Baseline demographic and clinical characteristics of the study participants (n = 80). Data are presented as mean ± standard deviation (SD) or n (%); 95% confidence intervals are provided where applicable. Ncm: Newton centimeter

Parameter		Value	95% confidence interval
Age (years)	Mean ± SD	32.45 ± 12.34	29.06-35.84
Sex, n (%)	Male	31 (38.7)	-
	Female	49 (61.3)	
Smoking, n (%)	Yes	12 (15.0)	
Implant torque at implant placement (Ncm)	Mean ± SD	67.98 ± 4.02	67.10-68.86
Periodontal depth at prosthetic loading (mm)	Mean ± SD	2.13 ± 0.47	2.03-2.24
Healing time at prosthetic loading (weeks)	Mean ± SD	13.04 ± 1.78	12.65-13.42

As shown in Table 3, implant stability increased progressively during the follow-up period. The mean ISQ improved from 67.98 ± 4.02 at implant placement to 70.76 ± 4.40 at prosthetic loading, 73.19 ± 4.45 at six months, and 74.28 ± 4.54 at 12 months. Repeated-measures ANOVA revealed a statistically significant difference across the evaluation periods (F = 32.96, p < 0.001). Bonferroni-adjusted pairwise comparisons (Table 4) showed significant improvements between implant placement and prosthetic loading, prosthetic loading and six months, six and 12 months, and implant placement and 12 months (all p < 0.004), indicating a continuous increase in implant stability over time.

**Table 3:** Comparison of implant stability quotient (ISQ) values at different time points. Data are presented as mean ± standard deviation (SD) with 95% confidence intervals; repeated-measures analysis of variance (ANOVA) was used for comparisons across time points. *p < 0.05 was considered statistically significant.

Time point	n	Mean ± SD (ISQ)	95% confidence interval	Repeated ANOVA (F)	p-value	Effect size (partial η²)
Implant placement (baseline)	80	67.98 ± 4.02	67.10-68.86	32.96	<0.001*	0.29
Prosthetic loading	80	70.76 ± 4.40	69.79-71.72			
6 months post-loading	80	73.19 ± 4.45	72.21-74.16			
12 months post-loading	80	74.28 ± 4.54	73.28-75.27			

**Table 4:** Bonferroni post hoc pairwise comparison of implant stability quotient (ISQ) values between time points. Data are presented as mean difference, Bonferroni-adjusted post hoc test following repeated-measures analysis of variance (ANOVA) was used, and Cohen's d represents effect size. *Bonferroni-adjusted statistical significance was set at p < 0.0125.

Comparison	Mean difference (ISQ)	t-value	p-value	Cohen's d
Placement vs loading	2.78	-13.54	<0.004*	1.51
Loading vs 6 months	2.43	-11.73	<0.004*	1.31
6 months vs 12 months	1.09	-6.31	<0.004*	0.71
Placement vs 12 months	6.30	-20.13	<0.004*	2.25

Radiographic evaluation revealed a gradual increase in marginal bone loss during the follow-up period (Table 5). The mean marginal bone loss increased from 0.19 ± 0.08 mm at prosthetic loading to 0.41 ± 0.13 mm at six months and 0.55 ± 0.16 mm at 12 months. Repeated-measures ANOVA demonstrated a significant difference between the three time points (F = 156.15, p < 0.001). Bonferroni post hoc analysis confirmed statistically significant differences between all pairwise comparisons (all p < 0.003) (Table 6).

**Table 5:** Comparison of radiographic marginal bone loss in mm at different time points. Data are presented as mean ± standard deviation (SD) with 95% confidence intervals; repeated-measures analysis of variance (ANOVA) was used for comparisons across time points. *p < 0.05 was considered statistically significant. mm: millimeters

Time point	n	Mean ± SD (mm)	95% confidence interval	Repeated ANOVA (F)	p-value	Effect size (partial η²)
Prosthetic loading	80	0.19 ± 0.08	0.17-0.20	156.15	<0.001*	0.66
6 months post-loading	80	0.41 ± 0.13	0.38-0.43			
12 months post-loading	80	0.55 ± 0.16	0.51-0.58			

**Table 6:** Bonferroni post hoc pairwise comparison of radiographic marginal bone loss. Data are presented as mean difference, Bonferroni-adjusted post hoc test following repeated-measures analysis of variance (ANOVA) was used, and Cohen's d represents effect size. *Bonferroni-adjusted statistical significance was set at p < 0.0167. mm: millimeters

Comparison	Mean difference (mm)	t-value	p-value	Cohen's d
Loading vs 6 months	0.22	-18.27	<0.003*	2.04
6 months vs 12 months	0.14	-16.46	<0.003*	1.84
Loading vs 12 months	0.36	-23.78	<0.003*	2.66

The peri-implant probing depth also exhibited a statistically significant increase during follow-up (Table 7). The mean probing depth increased from 2.13 ± 0.47 mm at prosthetic loading to 2.37 ± 0.58 mm at six months and 2.52 ± 0.61 mm at 12 months (F = 9.841, p < 0.001). Bonferroni-adjusted post hoc analysis demonstrated significant differences between all follow-up intervals (all p < 0.003) (Table 8).

**Table 7:** Comparison of peri-implant probing depth in mm at different time points. Data are presented as mean ± standard deviation (SD) with 95% confidence intervals; repeated-measures analysis of variance (ANOVA) was used for comparisons across time points. *p < 0.05 was considered statistically significant. mm: millimeters

Time point	n	Mean ± SD (mm)	95% confidence interval	Repeated ANOVA (F)	p-value	Effect size (partial η²)
Prosthetic loading	80	2.13 ± 0.47	2.03-2.24	9.841	<0.001*	0.11
6 months post-loading	80	2.37 ± 0.58	2.24-2.50			
12 months post-loading	80	2.52 ± 0.61	2.39-2.66			

**Table 8:** Bonferroni post hoc pairwise comparison of peri-implant probing depth. Data are presented as mean difference, Bonferroni-adjusted post hoc test following repeated-measures analysis of variance (ANOVA) was used, and Cohen's d represents effect size. *Bonferroni-adjusted statistical significance was set at p < 0.0167. mm: millimeters

Comparison	Mean difference (mm)	t-value	p-value	Cohen's d
Loading vs 6 months	0.24	-6.76	<0.003*	0.76
6 months vs 12 months	0.15	-5.08	<0.003*	0.57
Loading vs 12 months	0.39	-8.94	<0.003*	1.00

The incidence of postoperative complications remained low throughout the study (Table 9). No complications were observed in 71 (88.8%) implants at prosthetic loading, 69 (86.2%) implants at six months, and 64 (80.0%) implants at 12 months. Soft-tissue inflammation was observed in seven (8.8%), six (7.5%), and six (7.5%) implants at prosthetic loading, six months, and 12 months, respectively. Mild marginal bone loss was recorded in two (2.5%), three (3.8%), and six (7.5%) implants, respectively. Screw loosening occurred in 0 (0%), one (1.2%), and two (2.5%) implants. All 80 implants had complete measurements at the predefined assessment time points and were included in the repeated-measures analyses. Therefore, no missing-data imputation or last-observation-carried-forward procedure was required, and no participants were lost to follow-up. Overall, the findings demonstrated progressive improvement in implant stability following LLLT, while maintaining favorable peri-implant tissue health and a low frequency of biological and mechanical complications.

**Table 9:** Distribution of postoperative complications during follow-up. Data are presented as n (%).

Complication type	Loading, n (%)	6 months, n (%)	12 months, n (%)
None	71 (88.8)	69 (86.2)	64 (80)
Soft-tissue inflammation	7 (8.8)	6 (7.5)	6 (7.5)
Mild marginal bone loss (>0.5 mm)	2 (2.5)	3 (3.8)	6 (7.5)
Screw loosening	0 (0)	1 (1.2)	2 (2.5)
Implant failure/loss	0 (0)	0 (0)	0 (0)

## Discussion

This prospective observational study evaluated the influence of LLLT on implant osseointegration and peri-implant tissue health over a 12-month follow-up period. The findings demonstrated a progressive improvement in implant stability from implant placement to the final follow-up, accompanied by acceptable marginal bone remodeling, minimal increase in peri-implant probing depth, and a low incidence of biological and mechanical complications. Collectively, these observations suggest that adjunctive LLLT may positively influence healing dynamics around dental implants while maintaining favorable peri-implant tissue health.

A significant increase in ISQ values was observed throughout the study period. The greatest improvement occurred during the early healing phase, followed by continued improvement after prosthetic loading. This pattern reflects the biological transition from primary mechanical stability to secondary biological stability resulting from new bone formation and remodeling at the implant-bone interface. Photobiomodulation is believed to enhance mitochondrial activity, increase adenosine triphosphate synthesis, stimulate osteoblastic proliferation, and promote the expression of growth factors involved in bone regeneration [11]. These mechanisms accelerate mineralized tissue formation and improve bone-to-implant contact, thereby contributing to the progressive increase in implant stability observed in this study. Similar trends have been described in previous clinical investigations evaluating LLLT as an adjunctive therapy during implant healing [12].

Radiographic evaluation revealed a gradual increase in marginal bone loss during the observation period. However, the magnitude of bone remodeling remained within the range generally reported for conventionally healed implants. Early crestal bone remodeling is a physiological response associated with surgical trauma, establishment of the peri-implant soft-tissue seal, and functional adaptation following prosthetic loading [13]. Although the observed marginal bone changes were clinically acceptable, the contribution of LLLT to these findings cannot be isolated because the present study did not include a non-LLLT control group. The relatively limited bone loss observed in this study may be attributed to the anti-inflammatory and biostimulatory effects of LLLT, which have been shown to promote angiogenesis, reduce inflammatory mediator release, and enhance bone remodeling [8,14]. These effects may facilitate more favorable healing at the peri-implant interface, while minimizing excessive crestal bone resorption.

The peri-implant probing depth increased modestly over time but remained within clinically acceptable limits. Small increases in probing depth during the first year after implant loading are commonly associated with maturation of peri-implant soft tissues rather than pathological peri-implant disease. The low prevalence of soft-tissue inflammation and the maintenance of shallow probing depths indicated stable peri-implant mucosal health throughout the follow-up. LLLT has been reported to enhance fibroblast proliferation, collagen synthesis, and microvascular perfusion, thereby supporting soft-tissue maturation and wound healing [15]. These biological effects may have contributed to the favorable peri-implant soft-tissue response observed in the present investigation.

The incidence of postoperative complications was low throughout the study period. Most implants remained free from complications throughout the follow-up period, whereas implant failure and screw loosening occurred only in a small proportion of cases. These findings indicate that adjunctive LLLT did not adversely affect implant healing and may have contributed to the uncomplicated postoperative recovery [16]. Although this study was not designed to evaluate implant survival as the primary outcome, the low complication rate supports the safety and clinical feasibility of incorporating LLLT into routine implant therapy.

The findings of the present study are generally consistent with the growing body of evidence suggesting that LLLT can enhance early osseointegration and improve clinical healing following implant placement [16,17]. Nevertheless, variations among published studies continue to exist because of differences in the laser wavelength, output power, energy density, irradiation frequency, implant surface characteristics, surgical protocols, and methods used to assess implant stability. Such methodological heterogeneity makes direct comparisons challenging and underscores the need for standardized LLLT protocols in future clinical research.

Lobato et al. [18] evaluated LLLT in implants placed in fresh extraction sockets and observed an increase in implant stability during healing; however, no statistically significant difference was found between the laser and control groups. Similarly, Torkzaban et al. [19] reported significant improvements in implant stability over time in both laser and control groups without a significant intergroup difference. The progressive increase in ISQ observed in the present cohort is therefore consistent with the longitudinal improvement reported in these studies. However, because the present study lacked a non-irradiated control group, it cannot determine whether the observed increase represents an additional effect of LLLT beyond the physiological progression of osseointegration.

Clinical implications

Within the limitations of this study, adjunctive LLLT appears to be a simple, non-invasive, and clinically feasible approach for enhancing implant healing. Improved implant stability during the early healing phase may facilitate predictable osseointegration, potentially supporting early prosthetic rehabilitation in appropriately selected patients. Furthermore, the favorable peri-implant soft-tissue response and limited marginal bone remodeling observed in this study suggest that LLLT may be a useful adjunct in routine implant practice, particularly in patients for whom accelerated healing is desirable.

Limitations

The present study had several limitations. The absence of a parallel control group limits both the ability to establish a direct causal relationship between LLLT and the observed clinical changes and the ability to directly benchmark these outcomes against conventional implant healing. The observed increase in implant stability and magnitude of marginal bone remodeling were within ranges reported for conventionally healed, non-irradiated implants; therefore, these findings cannot be interpreted as an LLLT-specific effect. In addition, patient-reported outcomes, including postoperative pain, discomfort, and analgesic consumption, were not assessed despite the proposed analgesic and anti-inflammatory effects of LLLT. The relatively short follow-up period of 12 months did not permit assessment of long-term implant survival or peri-implant tissue stability. Furthermore, only one laser protocol was evaluated, precluding comparisons with alternative wavelengths or irradiation parameters. Future randomized controlled trials with larger sample sizes, appropriate control groups, patient-reported outcome measures, extended follow-up, and standardized LLLT protocols are warranted to determine whether LLLT provides additional clinical benefits beyond conventional implant healing.

## Conclusions

Adjunctive LLLT was accompanied by progressive improvement in implant stability, while marginal bone remodeling remained within clinically acceptable limits, peri-implant soft-tissue health remained favorable, and the incidence of postoperative complications was low during the 12-month follow-up after prosthetic loading. However, because this study lacked a non-LLLT or sham control group, these longitudinal changes cannot be attributed specifically to LLLT and may, at least in part, represent physiological osseointegration and functional bone remodeling. Controlled clinical trials with standardized laser protocols and longer follow-up periods are required to determine whether LLLT provides additional benefits beyond conventional implant healing.
